# Supplementary material for: Skeletal muscle stem cells modulate niche function in Duchenne muscular dystrophy mouse through YY1-CCL5 axis
Source: Nat Commun. 2025 Feb 3;16:1324. doi: 10.1038/s41467-025-56474-w (PMC11790879; doi:10.1038/s41467-025-56474-w)
Supplement: Supplementary file 1 — Supplementary Information [file 41467_2025_56474_MOESM1_ESM.pdf]

Inventory of Supplemental Information

Supplemental Figures

Suppl. Fig. 1. Inducible deletion of YY1 in MuSCs aggravates muscle dystrophy in mdx mouse.

Suppl. Fig. 2. Intrinsic deletion of YY1 in MuSCs alters cellular microenvironment in dystrophic muscle.

Suppl. Fig. 3. Intrinsic deletion of YY1 in MuSC induces enhanced crosstalk between MuSC and MP via CCL5/CCR5 axis.

Suppl. Fig. 4. TGFβ1 enriched niche inhibits FAP apoptosis and causes FAP accumulation in dKO muscle.

Suppl. Fig. 5. Targeting CCL5/CCR5 axis with MVC alleviates muscle dystrophy.

Suppl. Fig. 6. YY1 controls *Ccl5* expression in MuSC via regulating 3D looping interaction.

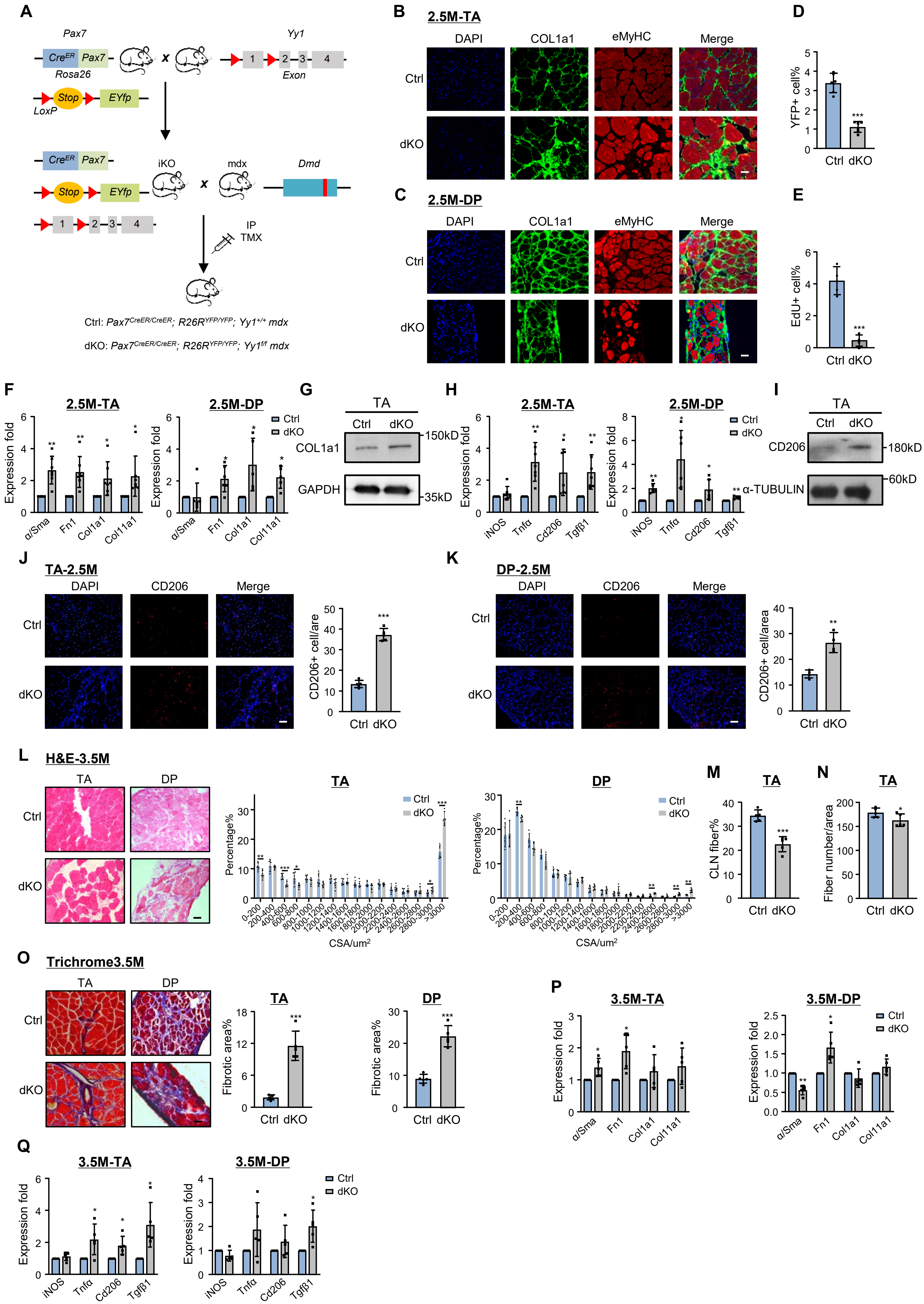

A
Pax7
CreER Pax7
Rosa26
Stop EYfp
LoxP
Yy1
1 2 3 4
Exon
iKO
mdx
Dmd
IP
TMX
Ctrl: Pax7CreER/CreER; R26RYFP/YFP; Yy1+/+ mdx
dKO: Pax7CreER/CreER; R26RYFP/YFP; Yy1f/f mdx
B
2.5M-TA
DAPI COL1a1 eMyHC Merge
Ctrl
dKO
C
2.5M-DP
D
YFP+ cell%
E
EdU+ cell%
F
2.5M-TA
2.5M-DP
Expression fold
α/Sma Fn1 Col1a1 Col11a1
G
TA
COL1a1
GAPDH
150kD
35kD
H
iNOS Tnfα Cd206 Tgfβ1
I
CD206
α-TUBULIN
180kD
60kD
J
TA-2.5M
CD206+ cell/are
K
DP-2.5M
CD206+ cell/area
L
H&E-3.5M
Percentage%
CSA/um2
M
CLN fiber%
N
Fiber number/area
O
Trichrome3.5M
Fibrotic area%
P
3.5M-TA
3.5M-DP
Q
3.5M-TA
3.5M-DP

Suppl. Fig. 1. Inducible deletion of YY1 in MuSCs aggravates muscle dystrophy in mdx mouse. **A** Breeding scheme for generating inducible YY1 conditional knock out (dKO) and Control (Ctrl) mdx mice. **B-C** IF staining of DAPI (blue), COL1a1 (green) and eMyHC (red) on the TA and DP muscles from 2.5M mice. Scale bar: 50 µm. **D** MuSC pool was detected in 2.5M Ctrl and dKO mice by flow cytometry (FC) with the YFP+ (Pax7+) signal, *n*=6 mice. *p*=0.0000021. **E** Quantification of EdU+ MuSCs isolated from 2.5M Ctrl and dKO mice, *n*=4 mice. *p*=0.00027. **F** RT-qPCR detection of fibrotic marker genes in TA and DP muscles from Ctrl and dKO mice, *n*=5 mice. *p*=0.0024, 0.0058, 0.030, 0.036. **G** Western blot detection of COL1a1 expression in the above TA muscles. **H** RT-qPCR detection of inflammatory marker genes in the above muscles. *n*=5 mice. *p*=0.43, 0.0033, 0.035, 0.0096. **I** Western blot detection of CD206 expression in the above TA muscles. **J-K** IF staining of DAPI (blue) and CD206 (green) on the above muscles and quantification of the number of CD206+ cells per area. Scale bar: 50 µm, *n*=5 mice. *p*=0.00000034 (J), 0.0011 (K). **L** H&E staining of TA and DP muscles from 3.5 M Ctrl and dKO mice. Distribution of fiber size is shown. Scale bar: 50 µm, *n*=5 mice. *p*=0.0056, 0.00046, 0.012, 0.034, 0.0000046 (TA); 0.0066, 0.0023, 0.0028, 0.0012 (DP). **M-N** Quantification of the fibers with centrally located nuclei (CLN) and the number in the above-stained muscles, *n*=5 mice. *p*=0.00014 (M), 0.034 (N). **O** Masson's Trichrome staining on the above muscles. Quantification of fibrotic areas is shown. Scale bar: 50 µm, *n*=5 mice. *p*=0.000056 (TA), 0.000033 (DP). **P** RT-qPCR detection of fibrotic marker genes in the above muscles, *n*=5 mice. *p*=0.035, 0.022, 0.30, 0.167. **Q** RT-qPCR detection of inflammatory marker genes in the above muscles, *n*=5 mice. *p*=0.30, 0.024, 0.014, 0.0099. All the bar graphs are presented as mean ± SD, paired two-sided Student's *t* test (F, H, P, Q) and unpaired two-sided Student's *t* test (D, E, J-N) were used to calculate the statistical significance: $*p < 0.05$, $**p < 0.01$, $***p < 0.001$, *n.s.* = no significance. Source data are provided as a Source Data file.

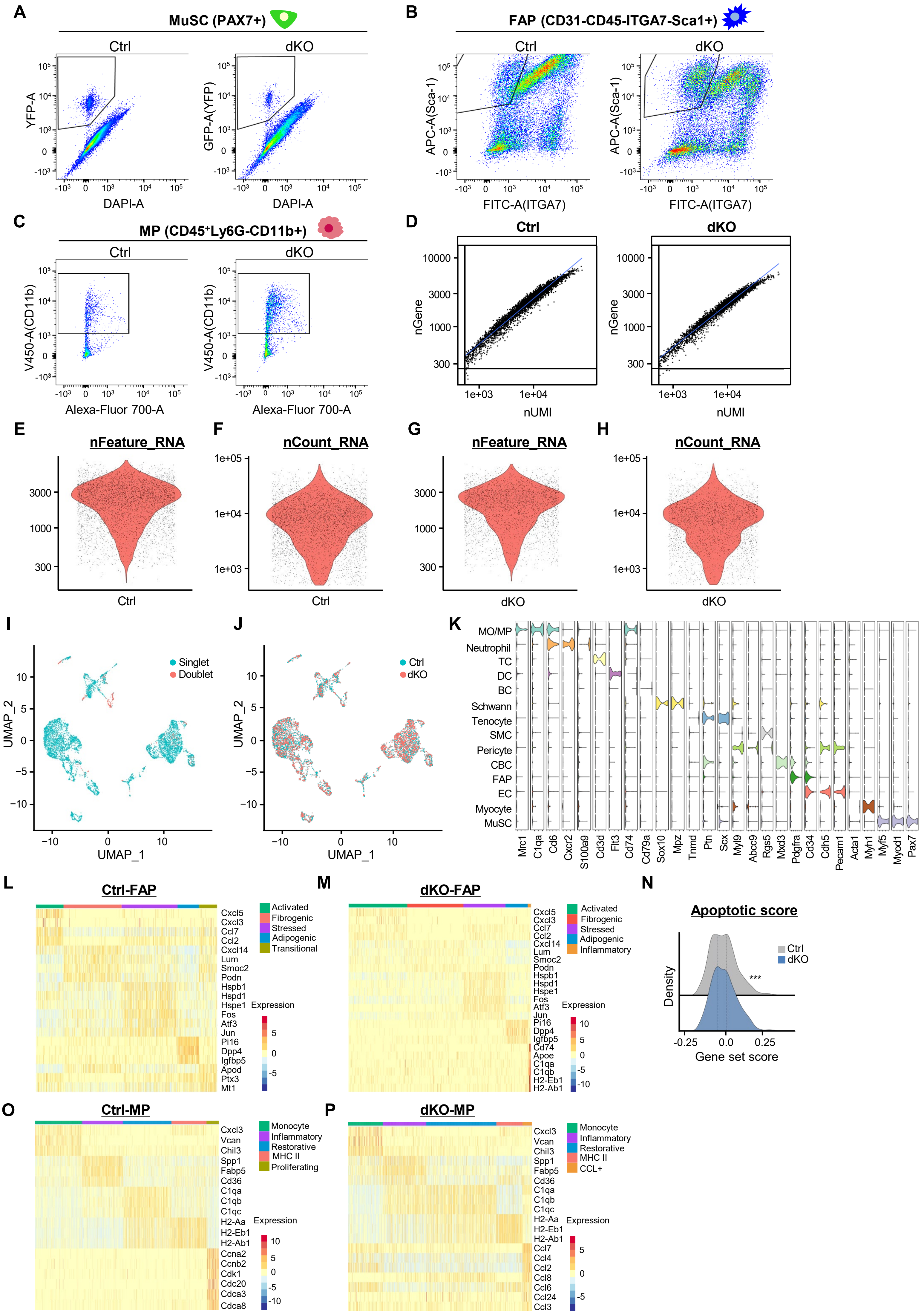

A
MuSC (PAX7+)
Ctrl
dKO
YFP-A
GFP-A(YFP)
DAPI-A
B
FAP (CD31-CD45-ITGA7-Sca1+)
APC-A(Sca-1)
FITC-A(ITGA7)
C
MP (CD45+Ly6G-CD11b+)
V450-A(CD11b)
Alexa-Fluor 700-A
D
nGene
nUMI
E
nFeature_RNA
F
nCount_RNA
G
nFeature_RNA
H
nCount_RNA
I
Singlet
Doublet
UMAP_2
UMAP_1
J
K
MO/MP
Neutrophil
TC
DC
BC
Schwann
Tenocyte
SMC
Pericyte
CBC
FAP
EC
Myocyte
MuSC
Mrc1
C1qa
Cd6
Cxcr2
S100a9
Cd3d
Flt3
Cd74
Cd79a
Sox10
Mpz
Tnmd
Ptn
Scx
Myl9
Abcc9
Rgs5
Mxd3
Pdgfra
Cd34
Cdh5
Pecam1
Acta1
Myh1
Myf5
Myod1
Pax7
L
Ctrl-FAP
Activated
Fibrogenic
Stressed
Adipogenic
Transitional
Expression
M
dKO-FAP
Inflammatory
N
Apoptotic score
Density
Gene set score
***
O
Ctrl-MP
Monocyte
Inflammatory
Restorative
MHC II
Proliferating
P
dKO-MP
CCL+

Suppl. Fig. 2. Intrinsic deletion of YY1 in MuSCs alters cellular microenvironment in dystrophic muscle. A-C Representative FACS plots of isolating MuSCs (YFP+), FAPs (FITC-, APC+) and MPs (FITC-, APC-, eFluor450+). About 100,000 cells were sorted by FACS from Ctrl and dKO mice at 7, 21 and 60 days post-TMX injection. The percentage of cells is shown in Fig 2B-D. **D** Scatter plots showing the correlation between the number of genes detected (nGene, X-axis) and the number of RNA counts (nUMI, Y-axis). **E-H** Violin plots showing the distribution of the number of features or fraction counts of detected RNAs in Ctrl and dKO muscles. **I** Uniform manifold approximation projection (UMAP) embedding of single-cell data showing the doublets removal. **J** UMAP embedding of single-cell data, colored by Ctrl or dKO. **K** Violin plots grouped by meta-clusters showing cell-type marker gene expression, which was used to classify clusters. **L-M** Heatmap of top genes as determined by FindAllMarkers on the subclustered FAPs in Ctrl and dKO. **N** Ridge map showing the global distribution density of apoptotic score of FAPs. p=0.000000000000035. **O-P** Heatmap of top genes as determined by FindAllMarkers on the subclustered MPs in Ctrl and dKO. Unpaired two-sided Student's t test (N) was used to calculate the statistical significance: $*p < 0.05$, $**p < 0.01$, $***p < 0.001$, n.s. = no significance. Source data are provided as a Source Data file.

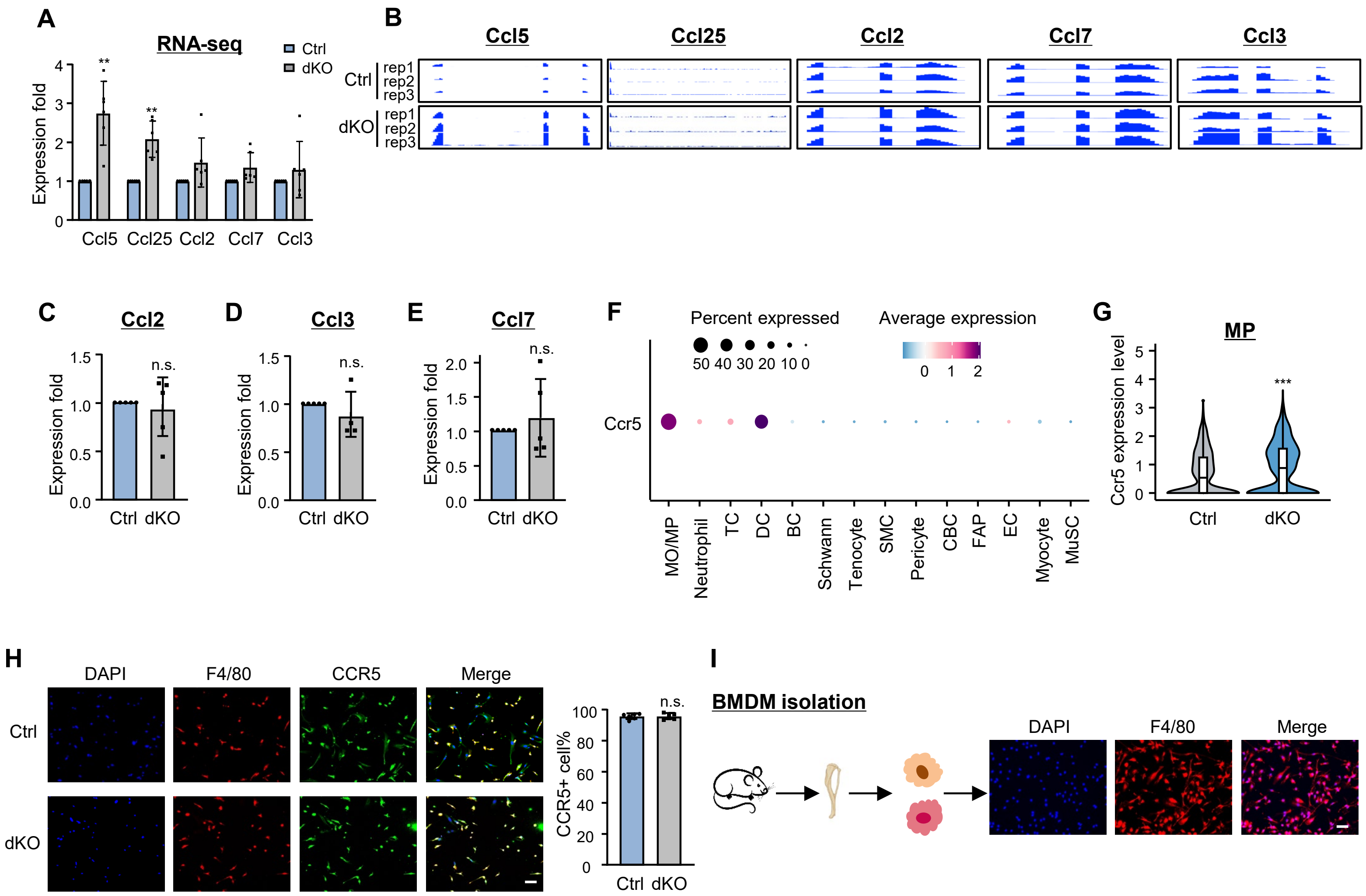

A
RNA-seq
Ctrl
dKO
Expression fold
Ccl5 Ccl25 Ccl2 Ccl7 Ccl3
B
Ccl5 Ccl25 Ccl2 Ccl7 Ccl3
Ctrl rep1 rep2 rep3
dKO rep1 rep2 rep3
C
Ccl2
D
Ccl3
E
Ccl7
n.s.
Ctrl dKO
F
Percent expressed
50 40 30 20 10 0
Average expression
0 1 2
Ccr5
MO/MP Neutrophil TC DC BC Schwann Tenocyte SMC Pericyte CBC FAP EC Myocyte MuSC
G
MP
Ccr5 expression level
Ctrl dKO
H
DAPI F4/80 CCR5 Merge
Ctrl
dKO
CCR5+ cell%
n.s.
I
BMDM isolation
DAPI F4/80 Merge

Suppl. Fig. 3. Intrinsic deletion of YY1 in MuSC induces enhanced crosstalk between MuSC and MP via CCL5/CCR5 axis. A Expression fold change of up-regulated *Ccl5*, *Ccl25*, *Ccl2*, *Ccl7*, *Ccl3* genes from RNA-seq data, n=6 mice. p=0.0035, 0.0023, 0.12, 0.073, 0.36. **B** Genomic snapshots of the above *Ccl* genes. **C-E** RT–qPCR validation of the expression of *Ccl2*, *Ccl3*, *Ccl7* genes in Ctrl and dKO MuSCs, n=5 (C, E) 4 (D) mice. p=0.68 (C), 0.21 (D), 0.48 (E). **F** Bar plot showing the *Ccr5* expression in cells analyzed by scRNA-seq data. **G** Volin plot showing the *Ccr5* expression in Ctrl and dKO MPs analyzed by scRNA-seq data. **H** Left: IF staining of DAPI (blue), F4/80 (red) and CCR5 (green) on isolated Ctrl and dKO MPs. Right: quantification of the percentage of CCR5+ cells. Scale bar: 50 µm, n=6. p=0.96. **I** Schematic of Bone Marrow Derived Macrophage (BMDM) isolation from *mdx* mouse and validation by F4/80 staining. Scale bar: 50 µm. All the bar graphs are presented as mean ± SD, paired two-sided Student's t test (A, C-E) and unpaired two-sided Student's t test (G, H) were used to calculate the statistical significance: *$p < 0.05$, **$p < 0.01$, ***$p < 0.001$, *n.s.* = no significance. Source data are provided as a Source Data file.

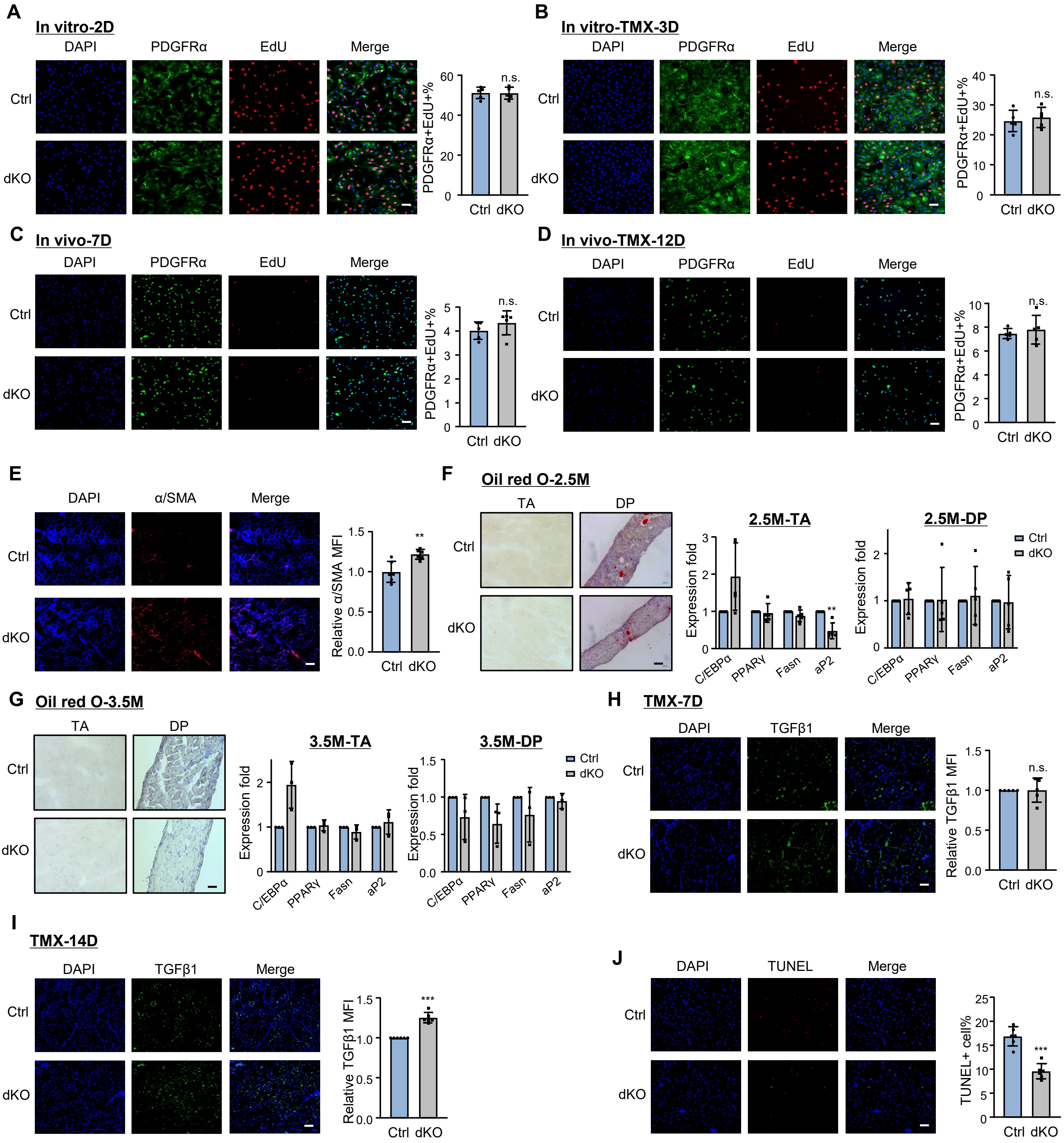

A
In vitro-2D
DAPI
PDGFRα
EdU
Merge
Ctrl
dKO
PDGFRα+EdU+%
n.s.
Ctrl dKO
B
In vitro-TMX-3D
DAPI
PDGFRα
EdU
Merge
Ctrl
dKO
PDGFRα+EdU+%
n.s.
Ctrl dKO
C
In vivo-7D
DAPI
PDGFRα
EdU
Merge
Ctrl
dKO
PDGFRα+EdU+%
n.s.
Ctrl dKO
D
In vivo-TMX-12D
DAPI
PDGFRα
EdU
Merge
Ctrl
dKO
PDGFRα+EdU+%
n.s.
Ctrl dKO
E
DAPI
α/SMA
Merge
Ctrl
dKO
Relative α/SMA MFI
**
Ctrl dKO
F
Oil red O-2.5M
TA
DP
Ctrl
dKO
2.5M-TA
2.5M-DP
Expression fold
Ctrl
dKO
C/EBPα
PPARγ
Fasn
aP2
G
Oil red O-3.5M
TA
DP
Ctrl
dKO
3.5M-TA
3.5M-DP
Expression fold
Ctrl
dKO
C/EBPα
PPARγ
Fasn
aP2
H
TMX-7D
DAPI
TGFβ1
Merge
Ctrl
dKO
Relative TGFβ1 MFI
n.s.
Ctrl dKO
I
TMX-14D
DAPI
TGFβ1
Merge
Ctrl
dKO
Relative TGFβ1 MFI
***
Ctrl dKO
J
DAPI
TUNEL
Merge
Ctrl
dKO
TUNEL+ cell%
***
Ctrl dKO

Suppl. Fig. 4. TGFβ1 enriched niche inhibits FAP apoptosis and causes FAP accumulation in dKO muscle. A-B FAPs were isolated from Ctrl and dKO muscles and cultured for 2 or 3 days and followed by EdU (red) and PDGFRα (green) staining. The percentage of EdU+ PDGFRα+ cells is shown. Scale bar: 50 μm, *n*=5 mice. *p*=0.94 (A), 0.60 (B). **C-D** FAPs were freshly isolated from Ctrl and dKO muscles 7 or 12 days post TMX injection and followed by EdU and PDGFRα staining. The percentage of EdU+ PDGFRα+ cells is shown. Scale bar: 50 μm, n=5. *p*=0.27 (C), 0.59 (D). **E** IF staining of DAPI (blue) and α/SMA (red) on 2.5M Ctrl and dKO TA muscles. Right: quantification of MFI of α/SMA. Scale bar: 50 μm, *n*=6 mice. *p*=0.0040. **F** Oil red O staining of the 2.5M TA and DP muscles from Ctrl and dKO mice. Scale bar: 50 μm. Right: RT-qPCR detection of adipogenic marker genes in TA and DP muscles from 2.5M Ctrl and dKO mice, n=5. *p*=0.081, 0.71, 0.17, 0.0049 (TA), 0.78, 0.93, 0.71, 0.92 (DP). **G** Oil red O staining of the 3.5M TA and DP muscles from Ctrl and dKO mice. Scale bar: 50 μm. Right: RT-qPCR detection of adipogenic marker genes in TA and DP muscles from 3.5M Ctrl and dKO mice. *n*=3 mice. *p*=0.27, 0.14, 0.38, 0.46. **H-I** Left: IF staining of TGFβ1 in TA muscles 7 days and 14 days after TMX administration. Scale bar: 50 μm. Right: quantification of MFI of TGFβ1, n=5. *p*=0.98 (H), 0.00022 (I). **J** FAPs were co-cultured with different numbers of Ctrl and dKO MPs, followed by TUNEL staining. The percentage of TUNEL+ cells is shown Scale bar: 50 μm, *n*=6 mice. *p*=0.000039. All the bar graphs are presented as mean ± SD, paired two-sided Student's *t* test (F, H, P, Q) and unpaired two-sided Student's *t* test (D, E, J-N) were used to calculate the statistical significance: **p* < 0.05, ***p* < 0.01, ****p* < 0.001, *n.s.* = no significance. Source data are provided as a Source Data file.

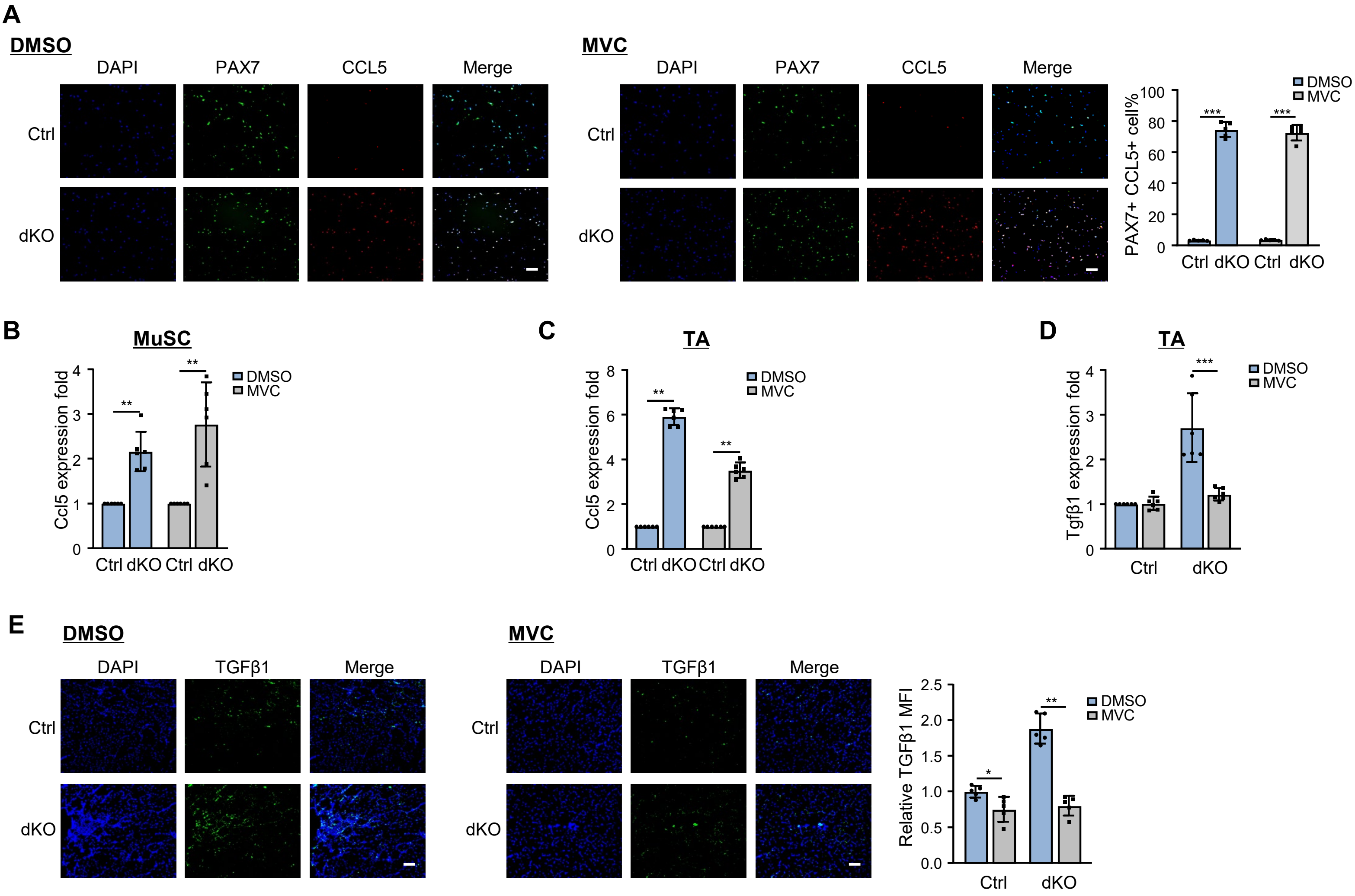
A
DMSO
DAPI
PAX7
CCL5
Merge
Ctrl
dKO
MVC
DAPI
PAX7
CCL5
Merge
Ctrl
dKO
PAX7+ CCL5+ cell%
DMSO
MVC
***
***
0
20
40
60
80
100
Ctrl dKO Ctrl dKO
B
MuSC
Ccl5 expression fold
DMSO
MVC
**
**
0
1
2
3
4
Ctrl dKO Ctrl dKO
C
TA
Ccl5 expression fold
DMSO
MVC
**
**
0
2
4
6
8
Ctrl dKO Ctrl dKO
D
TA
Tgfβ1 expression fold
DMSO
MVC
***
0
1
2
3
4
Ctrl
dKO
E
DMSO
DAPI
TGFβ1
Merge
Ctrl
dKO
MVC
DAPI
TGFβ1
Merge
Ctrl
dKO
Relative TGFβ1 MFI
DMSO
MVC
*
**
0.0
0.5
1.0
1.5
2.0
2.5
Ctrl
dKO

Suppl. Fig. 5. Targeting CCL5/CCR5 axis with MVC alleviates muscle dystrophy. A MuSCs were freshly isolated from DMSO and MVC treated mice and IF stained for DAPI (blue), PAX7 (green) and CCL5 (red). Scale bar: 50 μm. Right: quantification of the percentage of CCL5+ cells, n=6. p=0.00000000076, 0.0000000013. **B** RT-qPCR detection of *Ccl5* expression in the above cells, n=6 mice. p=0.0013, 0.0058. **C** RT-qPCR detection of *Ccl5* expression in TA and DP muscles from DMSO and MVC treated mice, n=6. p=0.00000054, 0.000010. **D** RT-qPCR detection of *Tgfβ1* expression in TA and DP muscles from DMSO and MVC treated mice, n=6 mice. p=0.0028, 0.0059. **E** IF staining of TGFβ1 in TA muscles from DMSO and MVC treated mice. Scale bar: 50 μm. Right: quantification of MFI of TGFβ1, n=6 mice. p=0.026, 0.0020. All the bar graphs are presented as mean $\pm$ SD, paired two-sided Student's t test (B-D) and unpaired two-sided Student's t test (A, E) were used to calculate the statistical significance: $^{*}p < 0.05$, $^{**}p < 0.01$, $^{***}p < 0.001$, *n.s.* = no significance. Source data are provided as a Source Data file.

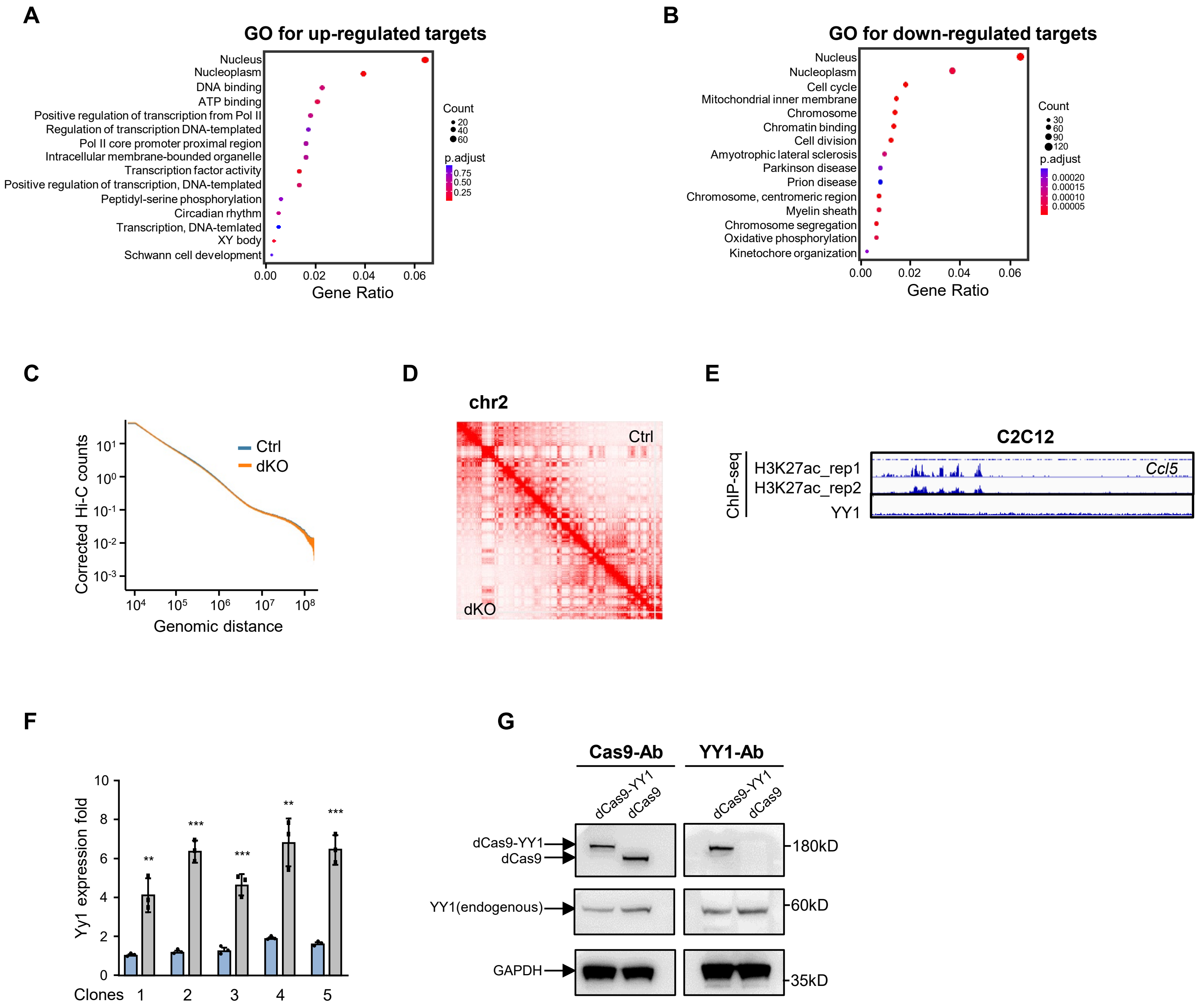
A
GO for up-regulated targets
Nucleus
Nucleoplasm
DNA binding
ATP binding
Positive regulation of transcription from Pol II
Regulation of transcription DNA-templated
Pol II core promoter proximal region
Intracellular membrane-bounded organelle
Transcription factor activity
Positive regulation of transcription, DNA-templated
Peptidyl-serine phosphorylation
Circadian rhythm
Transcription, DNA-temlated
XY body
Schwann cell development
0.00
0.02
0.04
0.06
Gene Ratio
Count
20
40
60
p.adjust
0.75
0.50
0.25
B
GO for down-regulated targets
Nucleus
Nucleoplasm
Cell cycle
Mitochondrial inner membrane
Chromosome
Chromatin binding
Cell division
Amyotrophic lateral sclerosis
Parkinson disease
Prion disease
Chromosome, centromeric region
Myelin sheath
Chromosome segregation
Oxidative phosphorylation
Kinetochore organization
0.00
0.02
0.04
0.06
Gene Ratio
Count
30
60
90
120
p.adjust
0.00020
0.00015
0.00010
0.00005
C
Corrected Hi-C counts
Ctrl
dKO
Genomic distance
D
chr2
Ctrl
dKO
E
C2C12
ChIP-seq
H3K27ac_rep1
H3K27ac_rep2
YY1
Ccl5
F
Yy1 expression fold
10
8
6
4
2
0
**
***
***
**
***
Clones 1 2 3 4 5
G
Cas9-Ab
YY1-Ab
dCas9-YY1
dCas9
dCas9-YY1
dCas9
dCas9-YY1
dCas9
YY1(endogenous)
GAPDH
180kD
60kD
35kD

Suppl. Fig. 6. YY1 controls *Ccl5* expression in MuSC via regulating 3D looping interaction. A-B GO analysis of overlapped up- or down-regulated genes in Fig. 6E. **C** Contact frequency as a function of genomic distance along the whole genome in Ctrl and dKO MuSCs. **D** Representative heatmap showing the called compartment at chromosome 2 in Ctrl (upper triangle) and dKO (lower triangle) MuSCs. **E** Genomic snapshots of H3K27ac and YY1 ChIP-seq tracks in C2C12 show no YY1 binding peak on *Ccl5* upstream enhancer sites. **F** RT-qPCR detection of overexpressed YY1 mRNAs in dCas9-YY1 vs. dCas9 transfected C2C12 cells, n=3 cells. P=0.0033, 0.000088, 0.00057, 0.0024, 0.00035. **G** Western blot validation of the expression of dCas9 or dCas9-YY1 in the above transfected C2C12 cells, the expression of endogenous YY1 protein is also shown. All the bar graphs are presented as mean ± SD, unpaired two-sided Student's t test (F), one-sided Fisher's exact test (A, B) were used to calculate the statistical significance and adjustments were made for multiple comparisons: $*p < 0.05$, $**p < 0.01$, $***p < 0.001$, *n.s.* = no significance. Source data are provided as a Source Data file.
