# Supplementary material for: Skeletal muscle stem cells modulate niche function in Duchenne muscular dystrophy mouse through YY1-CCL5 axis
Source: Nat Commun. 2025 Feb 3;16:1324. doi: 10.1038/s41467-025-56474-w (PMC11790879; doi:10.1038/s41467-025-56474-w)
Supplement: Supplementary file 3 — Description of Additional Supplementary Files [file 41467_2025_56474_MOESM3_ESM.pdf]

Description of Additional Supplementary Files

Supplementary Data 1. Sequences of oligonucleotides used in the study.

Supplementary Data 2. Single-cell RNA-seq profiling in Ctrl and YY1 dKO MuSCs.

Supplementary Data 3. Bulk RNA-seq in YY1 dKO MuSCs.

Supplementary Data 4. YY1 ChIP-seq in mdx MuSCs.

Supplementary Data 5. Hi-C analysis in Ctrl and YY1 dKO MuSC.
