# Supplementary material for: Skeletal muscle stem cells modulate niche function in Duchenne muscular dystrophy mouse through YY1-CCL5 axis
Source: Nat Commun. 2025 Feb 3;16:1324. doi: 10.1038/s41467-025-56474-w (PMC11790879; doi:10.1038/s41467-025-56474-w)
Supplement: Supplementary file 9 — Reporting Summary [file 41467_2025_56474_MOESM9_ESM.pdf]

nature portfolio

Corresponding author(s): Huating Wang

Last updated by author(s): Dec 5, 2024

Reporting Summary

Nature Portfolio wishes to improve the reproducibility of the work that we publish. This form provides structure for consistency and transparency in reporting. For further information on Nature Portfolio policies, see our Editorial Policies and the Editorial Policy Checklist.

Statistics

For all statistical analyses, confirm that the following items are present in the figure legend, table legend, main text, or Methods section.

n/a	Confirmed	
☐	☒	The exact sample size (*n*) for each experimental group/condition, given as a discrete number and unit of measurement
☐	☒	A statement on whether measurements were taken from distinct samples or whether the same sample was measured repeatedly
☐	☒	The statistical test(s) used AND whether they are one- or two-sided<br>*Only common tests should be described solely by name; describe more complex techniques in the Methods section.*
☒	☐	A description of all covariates tested
☒	☐	A description of any assumptions or corrections, such as tests of normality and adjustment for multiple comparisons
☐	☒	A full description of the statistical parameters including central tendency (e.g. means) or other basic estimates (e.g. regression coefficient) AND variation (e.g. standard deviation) or associated estimates of uncertainty (e.g. confidence intervals)
☐	☒	For null hypothesis testing, the test statistic (e.g. *F*, *t*, *r*) with confidence intervals, effect sizes, degrees of freedom and *P* value noted<br>*Give P values as exact values whenever suitable.*
☒	☐	For Bayesian analysis, information on the choice of priors and Markov chain Monte Carlo settings
☒	☐	For hierarchical and complex designs, identification of the appropriate level for tests and full reporting of outcomes
☐	☒	Estimates of effect sizes (e.g. Cohen's *d*, Pearson's *r*), indicating how they were calculated

Our web collection on statistics for biologists contains articles on many of the points above.

Software and code

Policy information about availability of computer code

Data collection	RT-qPCR were conducted using the Light Cycler ®480 Real-Time PCR System (Roche Applied Science);<br>Microscopy pictures were acquired with Leica microscope system DM 6000B; BD FACSVerse flow cytometer, BD FACSAria Fusion Cell Sorter and BD FACSDiva (version 8.0.1, BD Biosciences) were used to acquisition of flow cytometry data.
Data analysis	GraphPad Prism version 8.2 was used to data analysis.<br>Leica LASX software was used to acquisition and analysis of images from Leica microscope.<br>ImageJ 1.50i (National Institutes of Health) were used to quantification of Main fluorescence intensity.<br>Flowjo10.8.1 were used to acquire and analyze flow cytometry data.<br>The analysis associated with Bulk RNA-seq, ChIP-seq, Hi-C, scRNA-seq profiling were conducted mainly using custom code. Basic NGS-processing softwares including MACS2, HiCCUPs, HiC-Pro, Bowtie2, Topdom were used.<br>The code used in this study is available at Zenodo and the GitHub repository https://github.com/Hannah-bioinfo/Scripts_for_YY1_paper

For manuscripts utilizing custom algorithms or software that are central to the research but not yet described in published literature, software must be made available to editors and reviewers. We strongly encourage code deposition in a community repository (e.g. GitHub). See the Nature Portfolio guidelines for submitting code & software for further information.

Data

Policy information about availability of data

All manuscripts must include a data availability statement. This statement should provide the following information, where applicable:

- Accession codes, unique identifiers, or web links for publicly available datasets
- A description of any restrictions on data availability
- For clinical datasets or third party data, please ensure that the statement adheres to our policy

Bulk RNA-seq, YY1 ChIP-Seq, Hi-C, scRNA-seq data generated in this study have been deposited in Gene Expression Omnibus (GEO) database under the accession codes GSE250204. All other data supporting the findings of this study are available from the corresponding author on reasonable request.

Research involving human participants, their data, or biological material

Policy information about studies with human participants or human data. See also policy information about sex, gender (identity/presentation), and sexual orientation and race, ethnicity and racism.

Reporting on sex and gender	*Use the terms sex (biological attribute) and gender (shaped by social and cultural circumstances) carefully in order to avoid confusing both terms. Indicate if findings apply to only one sex or gender; describe whether sex and gender were considered in study design; whether sex and/or gender was determined based on self-reporting or assigned and methods used. Provide in the source data disaggregated sex and gender data, where this information has been collected, and if consent has been obtained for sharing of individual-level data; provide overall numbers in this Reporting Summary. Please state if this information has not been collected. Report sex- and gender-based analyses where performed, justify reasons for lack of sex- and gender-based analysis.*
Reporting on race, ethnicity, or other socially relevant groupings	*Please specify the socially constructed or socially relevant categorization variable(s) used in your manuscript and explain why they were used. Please note that such variables should not be used as proxies for other socially constructed/relevant variables (for example, race or ethnicity should not be used as a proxy for socioeconomic status). Provide clear definitions of the relevant terms used, how they were provided (by the participants/respondents, the researchers, or third parties), and the method(s) used to classify people into the different categories (e.g. self-report, census or administrative data, social media data, etc.) Please provide details about how you controlled for confounding variables in your analyses.*
Population characteristics	*Describe the covariate-relevant population characteristics of the human research participants (e.g. age, genotypic information, past and current diagnosis and treatment categories). If you filled out the behavioural & social sciences study design questions and have nothing to add here, write "See above."*
Recruitment	*Describe how participants were recruited. Outline any potential self-selection bias or other biases that may be present and how these are likely to impact results.*
Ethics oversight	*Identify the organization(s) that approved the study protocol.*

Note that full information on the approval of the study protocol must also be provided in the manuscript.

Field-specific reporting

Please select the one below that is the best fit for your research. If you are not sure, read the appropriate sections before making your selection.

☒ Life sciences ☐ Behavioural & social sciences ☐ Ecological, evolutionary & environmental sciences

For a reference copy of the document with all sections, see nature.com/documents/nr-reporting-summary-flat.pdf

Life sciences study design

All studies must disclose on these points even when the disclosure is negative.

Sample size	At least three biological replicates per group (detailed n is indicated in the figure or figure legends) were collected to perform statistical testing. No statistical test was used to determine sample size. For YY1 ChIP-seq profiling, MuSCs from 20 Ctrl mice were mixed and used. For scRNA-seq, 3 pairs of Ctrl and dKO mice were mixed and used. All the sample sizes determination were referred to our previous publications.
Data exclusions	No data or samples were excluded from the analysis
Replication	All experimental data was repeated in multiple biological independent experiments as indicated in the legend, method and source data.
Randomization	For each animal experiments, we randomly used the same age and sex of Ctrl and dKO mice from the same litter whenever possible. For cell experiments, we randomly counted multiple fields or cells per group for calculation.
Blinding	For all experiments, we performed the experiments in a blinded way.

Reporting for specific materials, systems and methods

We require information from authors about some types of materials, experimental systems and methods used in many studies. Here, indicate whether each material, system or method listed is relevant to your study. If you are not sure if a list item applies to your research, read the appropriate section before selecting a response.

Materials & experimental systems

n/a	Involved in the study
☐	☒ Antibodies
☐	☒ Eukaryotic cell lines
☒	☐ Palaeontology and archaeology
☐	☒ Animals and other organisms
☒	☐ Clinical data
☒	☐ Dual use research of concern
☒	☐ Plants

Methods

n/a	Involved in the study
☐	☒ ChIP-seq
☐	☒ Flow cytometry
☒	☐ MRI-based neuroimaging

Antibodies

Antibodies used	Antibodies of YY1 (Abcam, ab109237, 1:1000), α-Tubulin (Santa Cruz Biotechnology sc-23948, 1:5000), CCL5 (Abcam ab189841, 1:500), TGFβ1 (Abcam ab92486, 1:1000), GAPDH (Sigma-Aldrich G9545-100UL, 1:5000), CAS9 (CST 14697T, 1:1000), CD206 (Thermo Fisher PA5-46994, 1:500), Collagen1a1 (Novus Biologicals NB600-450, 1:500) were used for Western blot. Antibodies of PAX7 (Developmental Studies Hybridoma Bank; 1:50), YY1 (Abcam ab109237, 1:200), CCL5 (Abcam ab189841; 1:200), F4/80 (Abcam ab6640, 1:200), PDGFRα (R&D BAF1062; 1:200), Ki67 (Santa Cruz Biotechnology, sc-23900; 1:200) for IF staining of cells. Antibodies of CCL5 (Abcam ab189841; 1:200), CCR5 (Abcam ab65850; 1:200), TGF-β1 (Abcam ab92486; 1:200), PDGFRα (R&D BAF1062; 1:200), Collagen Ia1(Novus NBP1-30054; 1:200), F4/80 (Abcam ab6640), CD68 (Biorad MCA1957GA; 1:200), CD206 (Abcam ab64693;1:200), Laminin (Sigma-Aldrich L9393-100UL, 1:800), α-SMA (Invitrogen, 14-9760-82; 1:200), Ki67 (Santa Cruz Biotechnology sc-23900; 1:200); PAX7 (Developmental Studies Hybridoma Bank; 1:50), eMyHC (Leica NCL-MHC-d; 1:200) for staining of muscle cryosections. Antibody of YY1 (Santa Cruz Biotechnology, sc-1703) for ChIP-seq assay.
Validation	All antibodies used are commercially available and the applications have been tested by the manufacturer.

Eukaryotic cell lines

Policy information about cell lines and Sex and Gender in Research

Cell line source(s)	Mouse C2C12 myoblast cells (CRL-1772) and 293T cells were obtained from American Type Culture Collection (ATCC).
Authentication	Cell lines were procured from commercial source and therefore, not authenticated.
Mycoplasma contamination	All cell lines were tested as negative for mycoplasma contamination.
Commonly misidentified lines (See ICLAC register)	NA

Animals and other research organisms

Policy information about studies involving animals; ARRIVE guidelines recommended for reporting animal research, and Sex and Gender in Research

Laboratory animals	All animal handling procedures, protocols and experiments ethics approval was granted by the CUHK AEEC (Animal Experimentation Ethics Committee) under the Ref No. 21-080-GRF and 19-220-MIS. The mice were maintained in animal room with 12 h light/12 h dark cycles, temperature (22–24 °C), and humidity (40–60%) at animal facility in CUHK, fed with PicoLab® Select Mouse Diet 50 IF/9F Diet and provided with plenty of fresh clean water at all times. For all animal-based experiments, at least three pairs of littermates or age-matched mice were used. Pax7CreER (Pax7tm1(cre/ERT2)Gaka) were kindly provided by Dr. Shahragim Tajbakhsh. ROSAEYFP, Yy1f/f and C57BL/10 ScSn DMDmdx (mdx) mouse strains were purchased from The Jackson Laboratory. The YY1-inducible conditional KO (YY1iKO) strain (Ctrl: Pax7CreERT2/R26YFP; Yy1+/+, Yy1iKO: Pax7CreERT2/R26YFP; Yy1f/f mice) was generated by crossing Pax7CreERT2/R26YFP mice with Yy1f/f mice. The Yy1/mdx double KO (YY1dKO) strain (Ctrl: Pax7CreERT2/R26YFP; Yy1+/+; mdx, YY1dKO: Pax7CreERT2/R26YFP; Yy1f/f; mdx) was generated by crossing YY1iKO with mdx mice.
Wild animals	NA
Reporting on sex	For each the animal experiments, we used the the same age and sex of Ctrl and dKO mice from the same litter whenever possible.
Field-collected samples	NA

Ethics oversight	All animal handling procedures and protocols were approved by the Animal Ethics Committee at Chinese University of Hong Kong.

Note that full information on the approval of the study protocol must also be provided in the manuscript.

Plants

Seed stocks	*Report on the source of all seed stocks or other plant material used. If applicable, state the seed stock centre and catalogue number. If plant specimens were collected from the field, describe the collection location, date and sampling procedures.*
Novel plant genotypes	*Describe the methods by which all novel plant genotypes were produced. This includes those generated by transgenic approaches, gene editing, chemical/radiation-based mutagenesis and hybridization. For transgenic lines, describe the transformation method, the number of independent lines analyzed and the generation upon which experiments were performed. For gene-edited lines, describe the editor used, the endogenous sequence targeted for editing, the targeting guide RNA sequence (if applicable) and how the editor was applied.*
Authentication	*Describe any authentication procedures for each seed stock used or novel genotype generated. Describe any experiments used to assess the effect of a mutation and, where applicable, how potential secondary effects (e.g. second site T-DNA insertions, mosiacism, off-target gene editing) were examined.*

ChIP-seq

Data deposition

☒ Confirm that both raw and final processed data have been deposited in a public database such as GEO.

☒ Confirm that you have deposited or provided access to graph files (e.g. BED files) for the called peaks.

Data access links<br>*May remain private before publication.*	https://www.ncbi.nlm.nih.gov/geo/query/acc.cgi?acc=GSE250204<br>secure token: uhwhyowwpxghbmr
Files in database submission	GSE250204_RAW.tar
Genome browser session<br>(e.g. UCSC)	UCSC

Methodology

Replicates	1 YY1 ChIP-seq; 1 IgG ChIP. Due to the limited number of MuSCs from one mouse, we used the mixed MuSCs isolated from 20 Ctrl mdx mice for ChIP-seq assay.
Sequencing depth	total reads: 21518462, uniquely mapping reads:13357709, 150bp of raw reads, paired end
Antibodies	YY1 (Santa Cruz Biotechnology, sc-1703)
Peak calling parameters	Candidate peaks were compared with the background, dynamic programming was used to determine λ of Poisson distribution, and the P-value cutoff was set to 0.0001 for YY1 ChIP-Seq experiment.
Data quality	4681 peaks were called under cutoff p value <0.0001
Software	MACS2

Flow Cytometry

Plots

Confirm that:

☒ The axis labels state the marker and fluorochrome used (e.g. CD4-FITC).

☒ The axis scales are clearly visible. Include numbers along axes only for bottom left plot of group (a 'group' is an analysis of identical markers).

☒ All plots are contour plots with outliers or pseudocolor plots.

☒ A numerical value for number of cells or percentage (with statistics) is provided.

Methodology

Sample preparation	Muscle stem cell, FAPs and MPs isolation are described in the "Methods" section.
Instrument	BD FACSAria Fusion Cell Sorter

Software	Flowjo 10.8.1
Cell population abundance	Muscle satellite cells sorting:<br>We stained isolated cells by FACS with Pax7, PDGFRa, F4/80 antibody which are the marker of MuSC, FAPs, MPs and found 95% cells are positive.
Gating strategy	Muscle stem cell, FAPs and MPs gating strategies are described in the "Methods" section.

☒ Tick this box to confirm that a figure exemplifying the gating strategy is provided in the Supplementary Information.
